# Direct ink writing of high-resolution cellulose structures

**DOI:** 10.1038/s41598-023-49128-8

**Published:** 2023-12-12

**Authors:** Farnaz Rezaei, Daniel O. Carlsson, Jimmy Hedin Dahlstrom, Jonas Lindh, Stefan Johansson

**Affiliations:** 1 Department of Materials Science and Engineering, Uppsala University, 75105 Uppsala, Sweden; 2 Cytiva, Björkgatan 30, 753 23 Uppsala, Sweden

**Keywords:** Biomaterials, Techniques and instrumentation

## Abstract

3D printing is envisioned to play an important role in the production of membranes for e.g., water purification and bio-separation applications due to the prospect of creating new and cleverly designed structures. Among different 3D printing techniques, direct ink writing offers the opportunity to print a wide variety of materials with high-detail resolution. There is a range of parameters that need to be optimized in order to develop robust printing techniques at that scale. In this study, cellulose acetate (CA), which is a biocompatible material, has been used as an ink. In order to examine the printability and the possibility of printing features as small as a few µm, nozzles with different diameters and inks with varying amounts and molecular weights of CA were investigated. Findings in this study indicate that, depending on the wetting on the underlaying structure, the nozzle's internal and external diameter affects the detail resolution of the printed structure. Different inks result in different widths of printed strands and generally a higher amount and higher molecular weights of CA results in higher detail resolution. However, too high amount of CA and molecular weight will increase the clogging risk in the nozzle. In this study, the internal size of the nozzle was 3 µm, and by selecting a  suitable ink, it was possible to print strands down to 1 µm size and 6 µm inter-strand distance in the air, bridging supports with limited sagging. Furthermore, wall structures consisting of 300 layers, corresponding to about 300 µm in total height, were successfully printed.

## Introduction

3D printing, also known as additive manufacturing (AM), enables the fabrication of complex 3D structures through layer-by-layer deposition of materials on to a selected substrate based on computer-aided design models. Over the past decades, 3D printing has demonstrated its ability to produce structures with controlled and intricate geometries^1–3^. 3D printing offers several advantages over conventional manufacturing methods, and one of the significant is the possibility to manufacture structures of complex geometries. Additionally, reduced material usage and waste generation are other important general benefits of this technique.

AM has applications in various fields, including drug delivery, microfluidics, biosensors, and separation systems^2,4^. Currently, there is a considerable interest in the possibility of preparing membranes with more precise macro and microstructures^5^, for purposes such as wastewater treatment^6^, medical devices and bio-separation^7^, including membrane chromatography^8^. The demand for high-detail resolution is one of the factors that has so far limited the use of AM in membranes productions^5^. Several techniques exist for fabricating microscale structures via 3D printing, including two-photon polymerization (TPP)^5^, and direct ink writing (DIW)^9^. In the TPP method, two low-energy photons, synchronized in time and space, are employed to cross-link the liquid polymer with-in the focal spot, resulting in high spatial resolution. However, the limited number of resins available for TPP restricts its use for membrane production^10,11^.

In direct ink writing, which is a dispensing-based method, the desired material (ink) is deposited through a nozzle on to a substrate^12^. One of the main advantages of DIW is the diversity of printable materials, including polymers, hydrogels, ceramics, and even metals^11,13^. The latter two powder-based inks will require thermal postprocessing. In the DIW method, ink is dispensed from the nozzle and the solvent in the ink evaporates. Several parameters can alter the way that ink flows through the nozzle, impacting print control and quality. Factors such as the concentration, the chain entanglement and the molecular weight of the polymer influence the properties of the ink^14^. The possibility of strand formation increases with increasing molecular weight, greater chain entanglement and a larger amount of polymer in the solution, analogous to electrospinning^15^. During the printing process the optimum ink composition is crucial as inks with too high amount of CA or too high molecular weight can lead to clogging in the nozzle, while low amounts and low molecular weights of CA can result in discontinuous strands^16^. The ink composition and the size of the nozzles are factors which determine the resolution of the printed structure. As discussed previously^9^, it is possible to control the deposition speed with nozzle diameters as small as 0.5 µm for a polyelectrolyte ink.

Cellulose has attracted extensive research attention as a viable raw material for 3D printing applications due to its excellent performance^17^. In this study cellulose has been chosen partially since it is renewable, degradable, and the most abundant natural biopolymer^18–20^. Furthermore, cellulose is a bioactive, biodegradable and biocompatible material which make it suitable for our intended applications^21^. Besides bio separation applications, cellulose also has a broad area of use, in for instance biomedical and pharmaceutical applications^22,23^. Various types of cellulose-based chromatography adsorbents e.g. monoliths, microspheres, and membranes are available^24^. In this area, membrane chromatography can be used for purification of e.g. biological macromolecules and is an efficient alternative to traditional column chromatography^25^. One of the challenges with cellulose is its insolubility in water and other common organic and inorganic solvents. To enhance its solubility, derivatives of cellulose can be used. There are different cellulose derivatives, and among them cellulose acetate (CA) stands out as the most promising^26^. CA is an acetate ester of cellulose and it dissolves in many solvents^27^. Previous studies has used nozzles ranging from 150 µm to a few mm in diameter for cellulose-based inks^28–30^. In this work, we will explore the use of single digit µm nozzle diameters to 3D print various test structures with high detail resolution.

In this study, our aim was to investigate the phenomena that control the detail resolution of membrane structures, printed using the DIW technique. The influence of nozzle dimensions, in particular inner and outer diameters, were examined to study the possibility of printing structures with µm-level detail resolution. We provide a detailed discussion of various factors, like wetting properties, ink composition, and nozzle diameter, affecting the printing process.

## Experimental

### Nozzle preparation

**Figure 1:**
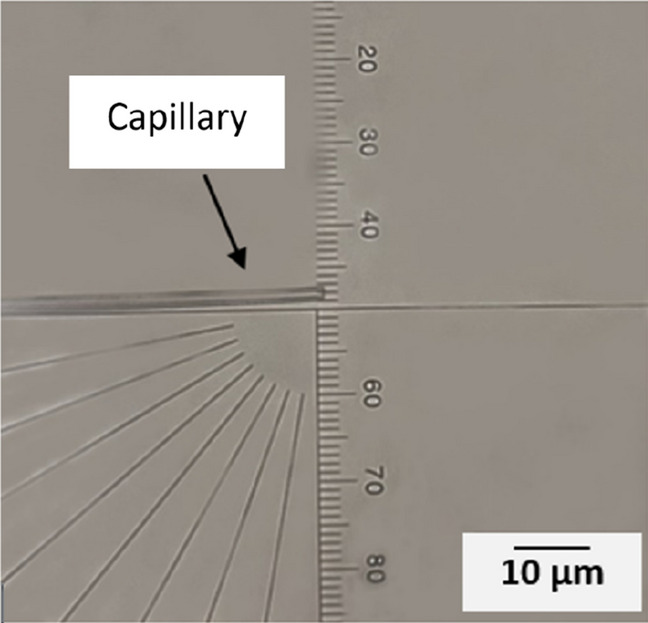
View of the capillary in the microforge. The left side of the picture shows the capillary. The distance between each mark on the scale is 3 µm.

Nozzles with diameters in the range of few micrometer diameters were prepared to evaluate the feasibility of 3D printing structures with detail resolution in the micron range. For preparing the nozzles, borosilicate glass capillaries were pulled with a micropipette puller (P-1000, Sutter instruments). The original internal and external diameters of the capillaries before pulling are 0.58 mm and 1 mm, respectively. Parameters like heat, pull and velocity, (these numbers represent the internal configuration of the micropipette puller machine), were adjusted to control the size and shape of the taper, Table 1. After pulling, a microforge (Narishige MF-900) was used to cut and reshape the capillaries into the desired size and shape. The microforge is equipped with a platinum wire and by attaching a glass bead to this wire and adjusting the wire's temperature, cutting and shaping the capillary becomes possible. Both inner and outer dimensions of the tapered capillary nozzle can be adjusted. The distance between the glass bead and the capillary is an essential parameter in the shaping process. Figure 1 depicts a capillary nozzle with an outer diameter of 6 µm (the distance between every two lines is 3 µm). Capillaries with the desired shape and size were glued to the syringe needles with fast-curing epoxy, to reduce the risk of leakage they were heat-treated for 7–8 min at 140 $^\circ{\rm C}$. Capillaries were coated with Trichloro silane (97%) from Sigma–Aldrich by placing them in a vacuum chamber next to 20 µl of silane for 24 h.

**Table 1:** Program settings in the micropipette puller, Sutter instruments, for pulling the glass capillaries. The numbers represent the internal settings of the machine and have no directly translatable units.

Program	Heat	Pull	Velocity	Time	Pressure	Ramp
Capillary with short taper	475	0	42	250	500	477
Capillary with long taper	459	0	150	0	500	476

### Ink formulation

As mentioned above and will be discussed, the selection of the molecular weight and amount of the CA are two of the most important parameters in the printing process. In this work, CA with three different molecular weights (30, 50, and 100 kDa), purchased from Sigma–Aldrich, and varying amounts of CA (5%, 7.5%, 10%, 12.5% and 15%) were examined. The inks were prepared by mixing CA with different amounts of solvent [Acetone, Acetone/ Dimethyl sulfoxide (DMSO) (1:1), Acetone/ Ethanol (1:1), or Acetone/Glycerol (5, 1)] at room temperature. The solutions were stirred for approximately 24 h until they became transparent.

### Filling the syringe with ink

To reduce the risk of contamination, the syringe was rinsed with acetone and milli-Q water before filling it with ink. The syringe was loaded with ink, using cellulose acetate filters with a pore size of 0.45 µm. Subsequently, the syringe filled with ink was centrifuged for 5 min at 1500 g to reduce the air bubbles inside the solution.

### Printing setup

**Figure 2:**
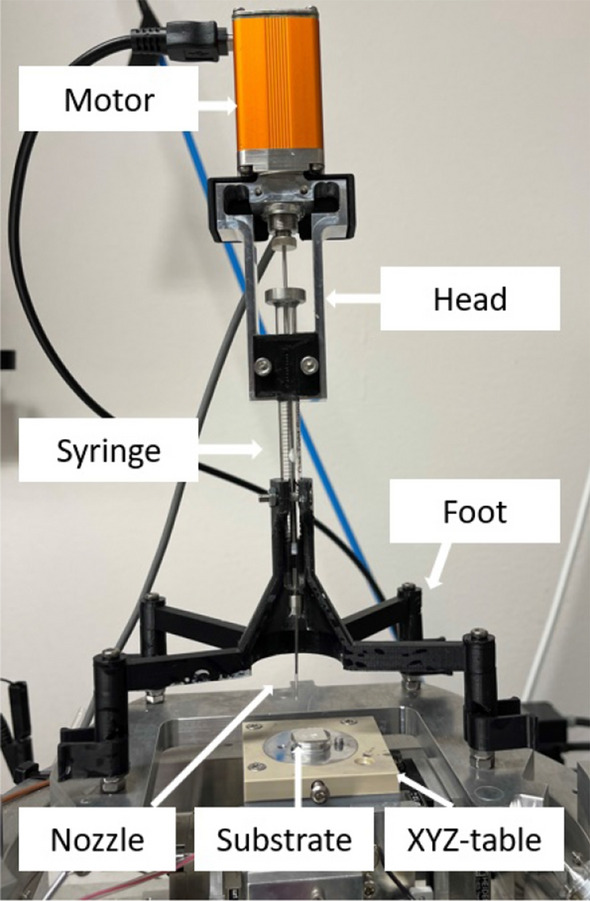
Set-up of the direct ink writing printer.

Figure 2 shows the experimental setup used in this study. This setup was developed in-house previously. At the bottom of the image, the stage can move in X, Y, and Z directions, providing an approximate range of 25, 25 and 10 mm, respectively. Uncoated glass slides measuring 1 × 1 cm were attached to scanning electron microscopy (SEM) stub holders using liquid silver and mounted on the stage. LabView software was used to control the printing process with a program developed in-house. This software enables precise control of the substrate’s position in the X, Y and Z directions. Prior to printing, programmed trajectories were uploaded to the software, guiding the movement of the substrate table. On top of the substrate, the syringe was fixed in an in-house 3D printed holder. The syringe’s piston is in contact with the drive rod of a linear motor, Piezomotor LTC2014. When the motor rod presses on the piston, the ink flows through the nozzle on to the substrate. A video camera with $\times$ 150 magnification was placed in front of the setup to monitor the printing process. In this study, the substrate’s speed, hence the printing speed, in the X and Y direction was set at 0.5 mm/s. The distances between each printed layer in the Z direction ranged from 0.5 to 1 µm, depending on the specific ink used in the experiment. The initial distance between the nozzle and the glass substrate was fixed at approximately 1 µm. The attached syringe was filled with different inks in each experiment, and the ink was dispensed with a linear motor rod unloaded speed corresponding to ink flow rates within a range from 100 to 1000 pl/s.

### Printing patterns

**Figure 3:**
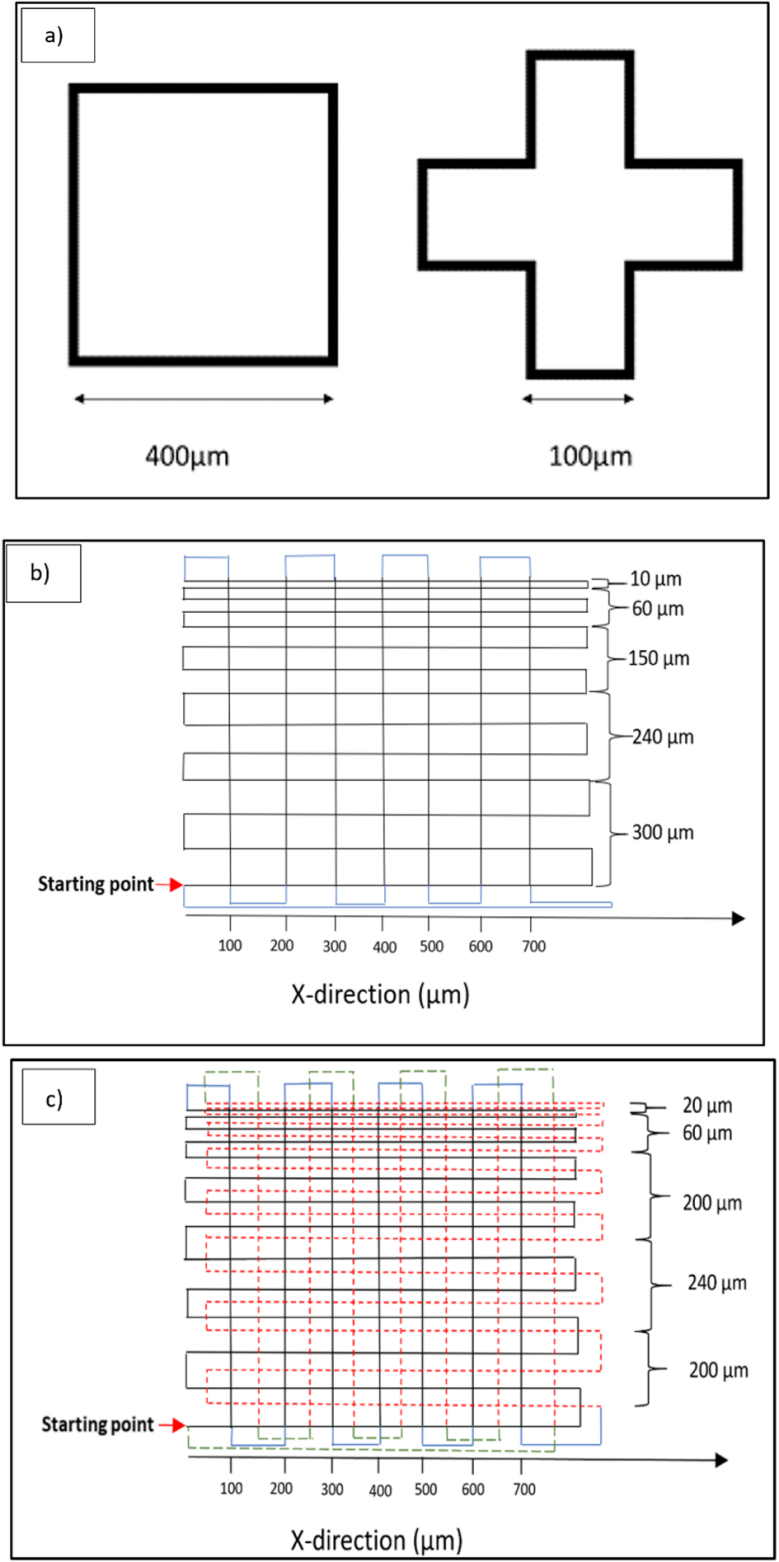
(**a**) Structure one; cubic and cross shaped structures (**b**) structure two; parallel lines in a rectangular pattern (**c**) Structure three; shift between layers.

**Figure 4:**
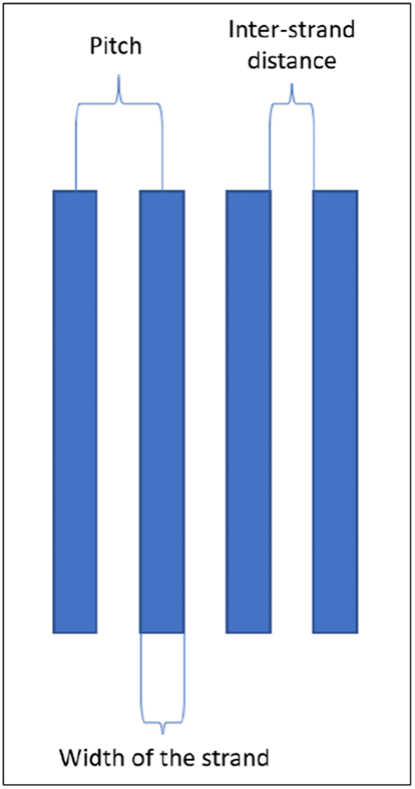
The illustration shows the pitch, inter-strand distance, and width of the strand.

Three different patterns of structures were printed. The first patterns were test structures; cubic, and cross shape structures, shown in Fig. 3a. Figure 3b shows a second pattern, where the separation in the X-direction between lines is constant at 100 µm. The separation between every two printed lines in the Y-direction starts at 100 µm and, step-by-step, decreases to 80, 50, 30, and 10 µm, and the blue lines were printed to change direction between X and Y. Multiple layers were printed following the same pattern. The third pattern (Fig. 3c), employed the same base pattern as in Fig. 3b. However, in this case, the second layer was shifted in both the X and Y directions relative to the first layer. The black lines were printed first, and then a second layer with a 500 nm height difference was printed, represented by red dashed lines. For multi-layer structures, the first layer and every odd-numbered layer there after followed the same pattern, while the second layer and every even-numbered layer after that followed the shifted pattern. The distance between each line has been indicated in the image, and green dashed lines were printed for changing the direction in the X and Y directions in the shifted layer. Figure 4 shows the design indicating the width, pitch and inter-strand distance.

### Characterization

The contact angle of each of the inks was measured on uncoated, silanized and CA-coated glass substrates using a contact angle meter (Dataphysics OCA 15). Printed samples were coated with an Au/Pd target using a Polaron sputter to be investigated by scanning electron microscopy (SEM) (LEO 1550, Zeiss).

## Results and discussion

### Effect of capillary

**Figure 5:**
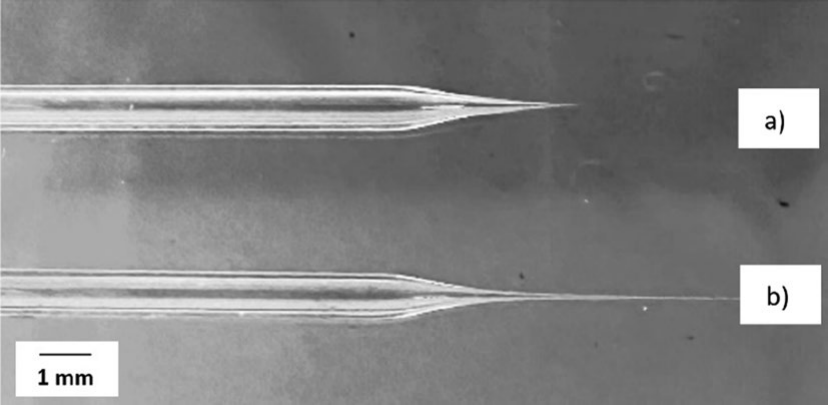
Glass capillaries that were prepared with different pulling parameters. (**a**) Capillary with a short taper. (**b**) Capillary with a long taper.

Figure 5 depicts capillaries with both short and long tapers, which were prepared by adjusting the different parameters outlined in Table 1. Capillaries with short tapers were prone to breaking during the printing process. To mitigate this issue, capillaries with longer tapers were used for printing the structures in this study. While the long taper capillaries are flexible and less prone to breaking compared to those with short tapers, they may introduce challenges related to reduced positioning control due to friction or pulling forces.

**Figure 6:**
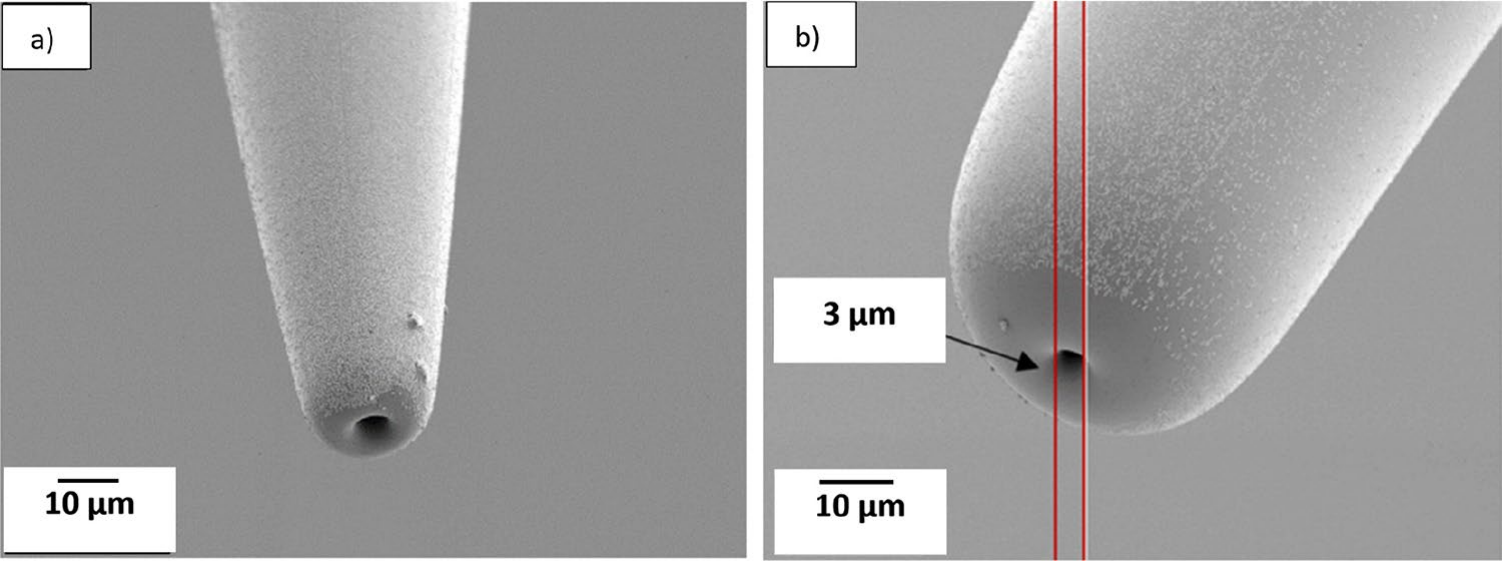
(**a**) SEM image of the capillary with an internal diameter of 3 µm and external diameter of 9 µm (3–9 capillary). (**b**), capillary with an inner dimension of 3 µm and an external dimension of 25 µm (3–25 capillary).

**Figure 7:**
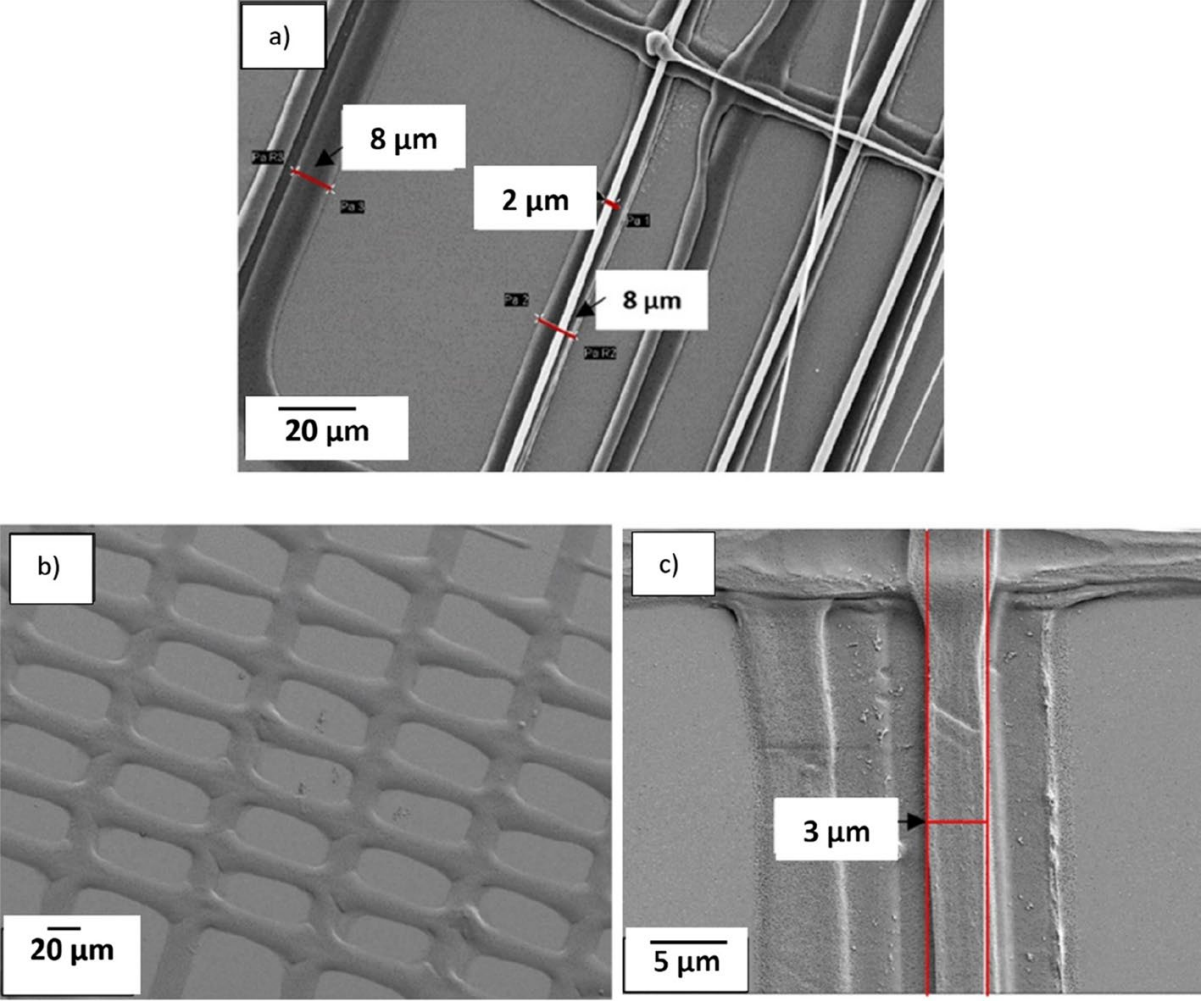
SEM image of the 3D Printed structure with ink containing 10% CA on the glass substrate with a 3–9 capillary. (**a**) Width of the printed strands in pattern two is 8 µm in the first layer and it reduce to two µm on a top layer. (**b**) The first layer and (**c**) the top layer of a structure printed with a 3–9 capillary. The strand placement varies between layers and it is most probably caused by capillary bending due to wetting force.

The effect of printing with capillaries having different diameters was studied by printing 10% CA ink on an uncoated glass substrate. Figure 6 shows the capillaries, which have an internal diameter of 3 µm and external diameters of about 9 µm (from here on named 3–9 capillary) or 25 µm (3–25 capillary). These capillary diameters were selected as reference dimensions to investigate various phenomena. In Fig. 7a, a 3–9 capillary was used to print pattern two (as defined in Fig. 3b). The width of the printed strand is almost identical to the external diameter of the nozzle in the first printed layer, and the width of the printed strand decreased to about 3 µm in the upper layers, which agrees with the internal diameter of the capillary used in this experiment. Figure 7b, also from printing pattern two, indicates that the width of the printed line is approximately 25 µm which matches the external diameter of the 3–25 capillary that was used for this print. In the top layer, the width decreased to about 3 µm, Fig. 7c, which agrees with the internal diameter of the capillary. Comparing two printed structures with the same design, and using the two mentioned capillaries indicates that the size of the printed structure depends on both the internal and external diameter of the capillary. In summary, Table 2 shows the capillaries with different diameters that has been used in this experiment and the size of the printed structures with each of the capillaries.

**Table 2:** Internal and external effect of the capillary on the width of the printed line in different layers.

Capillary	Internal diameter (µm)	External diameter (µm)	Width of the printed lines of the first layer (µm)	Width of the printed lines of the top layer (µm)
3–25	3	25	24–27	2–4
3–9	3	9	8–10	2–4

### Ink properties and performance

Different ratios of acetone, ethanol, glycerol, and DMSO were evaluated to investigate the printability and resolution that can be achieved. CA was dissolved in acetone, but did not completely dissolve in ethanol or glycerol. Also, both acetone and ethanol evaporate quickly and using solely these solvents typically causes clogging of the capillary. DMSO was therefore added to the acetone to reduce the rate of solvent evaporation. Different ratios of acetone and DMSO were examined, and the one with a 1:1 ratio of acetone to DMSO caused less clogging of the capillary than the other ratios of these solvents, and was selected for this study. Different amounts of cellulose acetate (CA) (5, 7.5, 10, 12.5, and 15%) with different molecular weights (30, 50, and 100 kDa) were examined, and the results are presented color-coded in Table 3. Inks with 12.5% and 15% CA and with a higher molecular weight solidified quickly after being dispensed from the nozzle and could cause clogging of the capillary after a few layers. Inks with 5% and 7.5% CA solidified slower compared to inks with 12.5% and 15% CA, and when printing the second layer of the structure, the first layer was not completely solidified. Printing large structures or structures with many layers is time-consuming and increases the risk of clogging. Increasing the amount of the CA to 10% and above made it possible to print thinner strands.

**Table 3:**
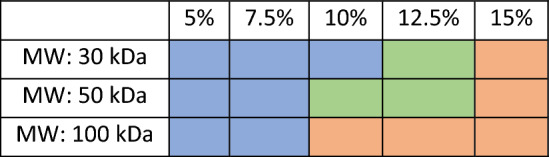
Inks with different molecular weight and concentration of CA using 1:1 acetone and DMSO as solvent. Color codes, blue: reduced detail resolution with rare clogging, green: high detail resolution and rare clogging problem, orange: high detailed resolution with frequent clogging.

**Figure 8:**
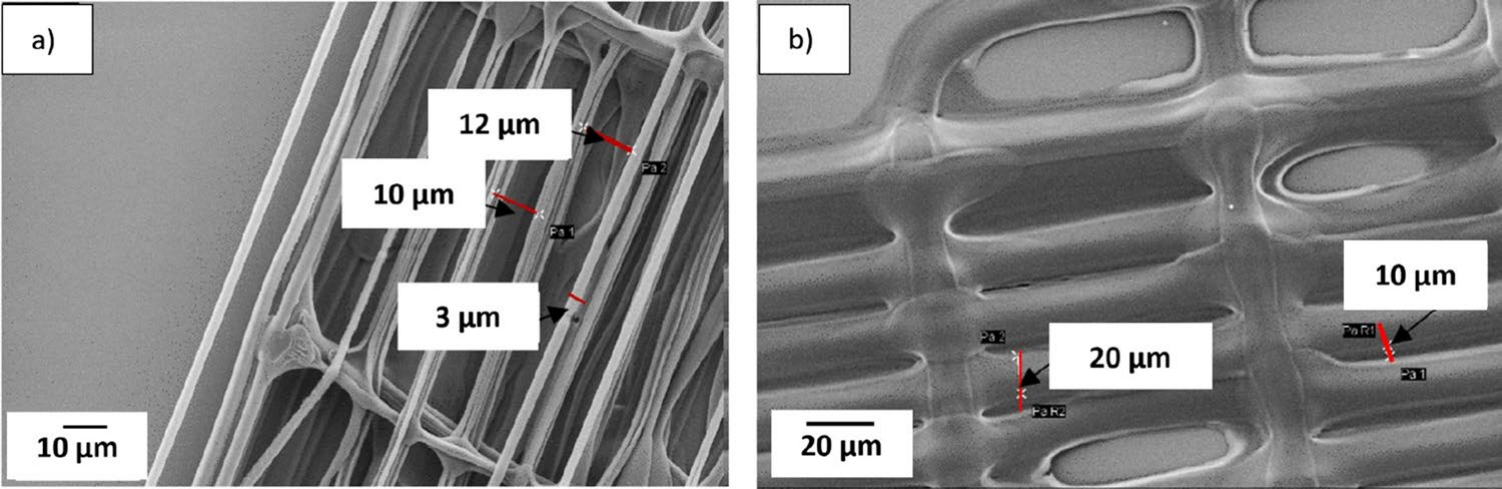
SEM micrographs showing the difference in width of the printed fibers by changing the amount of CA in inks, (**a**) Ink with 15% CA (50 kDa) was used resulting in a strand width of 3 µm, (**b**) Ink with 5% (50 kDa) CA resulting in a strand width of 20 µm Both structures are printed with 3–9 capillaries.

Figure 8, depicts structures with pattern number three, printed using 3–9 capillaries. In this experiment two inks with different amounts of CA were evaluated. Figure 8a indicates that the width of the printed structure with 15% CA (50 kDa) ink is close to the internal diameter of the capillary, 3 µm, and in Fig. 8b the width of the fiber is almost two times bigger than the external diameter of the capillary using 5% CA (50 kDa) ink.

## Mechanisms of controlling the size of the printed structures

To study factors that control the size of the printed strands, the wettability of inks with different amount and different molecular weights of CA was examined on both non-coated, silanized and CA-coated glass substrates, Table 4. The measurments for 15% CA with different molecular weights (30 kDa, 50 kDa, and 100 kDa) indicate an increased contact angle with increasing molecular weight. Comparing of inks with 7.5% and 15% CA, while keeping the molecular weight constant (50 kDa) suggests that the contact angle increases with an increased amount of CA. A silanized glass substrate was also evaluated and the contact angle of the ink with 15% CA and molecular weight of 50 kDa on the coated substrate was measured to 108° ± 8°. In comparison, the contact angle of the same ink on an uncoated substrate was 89° ± 10° which is in agreement with a decreased wetting by silane coating on the capillary, as desired. However, the large scatter in the contact angle measurements, partially due to the fast evaporation of the ink solvent, makes it difficult to draw safe conclusions. Although a larger contact angle of a silanized substrate would make it possible to print narrower structures in the first layer, this first CA layer of a structure does not attach sufficiently to the silanized substrate and makes it very hard to print a second layer. For this reason, all the print experiments in this study were made on uncoated glass substrates. Due to the complexity of measuring the contact angle of CA strands on top of the already printed layer, a complete coating of CA (15% and with molecular weight of 50 kDa) was applied onto the glass substrate. The results show that the contact angle of the ink with 15% CA (50 kDa) was larger for a CA-coated substrate in comparison with an uncoated substrate. As this coating forms a rough layer, and increases the risk of nozzle breakage, it was not used as a substrate coating. The reduced wetting of the ink on the CA coated substrate in comparison with glass would to a certain extent explain the reduced orthogonal spreading of the inks in the upper layers. Apart from factors such as solvent absorption in the substrate, the wall width at a certain layer height will also be a major factor controlling the width of the subsequent printed layer since the ink cannot spread further sideways.

**Table 4:** Contact angle of inks with different amounts and molecular weight of CA on glass (uncoated), silanized glass and CA coated substrates. The contact angle values are averages ± standard deviations for three different measurements.

Ink	15% CA (30 kDa)	15% CA (50 kDa)	15% CA (100 kDa)	7.5 CA (50 kDa)	15% CA (50 kDa)	15% CA (50 kDa)
Contact angle	79° ± 12°	89° ± 10°	114° ± 10°	63° ± 13°	108° ± 8°	107° ± 18°
Substrate	Uncoated	Uncoated	Uncoated	Uncoated	Silanized	Coated with CA

**Figure 9:**
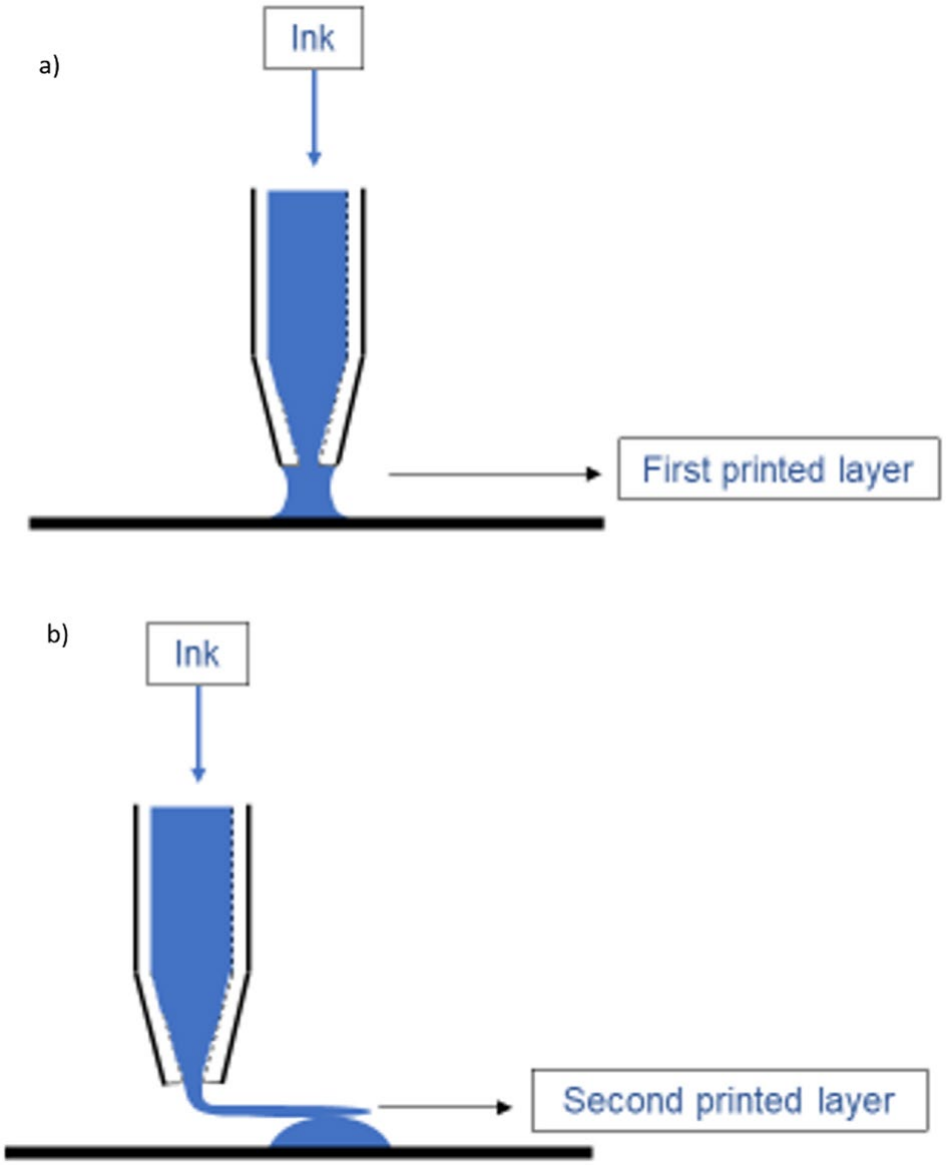
Size of the printed structure in the first layer (**a**) is typically controlled by the outer diameter of the capillary when there is some wetting on the substrate, and (**b**) internal diameter of the capillary typically controls the size of the printed structure in the subsequent layers when there is no or limited wetting on the printed structure.

In direct ink writing the wetting between the ink and surfaces as well as the chain entanglement of the ink will influence the printing process. As discussed, both the internal and external diameter of the nozzle (Table 2) affect the size of the printed strand. When printing the first layer the wettability of the substrate has a major effect and, for instance, if the ink wets the substrate better than the capillary, the size of the printed fiber is still typically controlled by the external diameter of the capillary, Fig. 9a. When printing the subsequent layers, where there is no, or little, influence from the substrate wetting effect, the reduced wetting on a silane coated nozzle should reduce the spreading of the ink from the outer to the inner capillary diameter. It also appears that the already printed strand is helping to pull out the strand from the syringe due to chain entanglement, and the size of the fiber is rather controlled by the internal diameter of the capillary, Fig. 9b. Furthermore, it is expected that the effect of the chain entanglement, as well as reduced wetting are more pronounced in the inks with higher amount and molecular weight of polymer. Figure 8a,b support a combination of a wetting and entanglement mechanisms, and shows that an ink with 5% CA will wet and spread laterally more easily than an ink with 15% CA which forms fine strands with a width close to the inner diameter of the capillary in the upper layers.

### Testing different patterns

**Figure 10:**
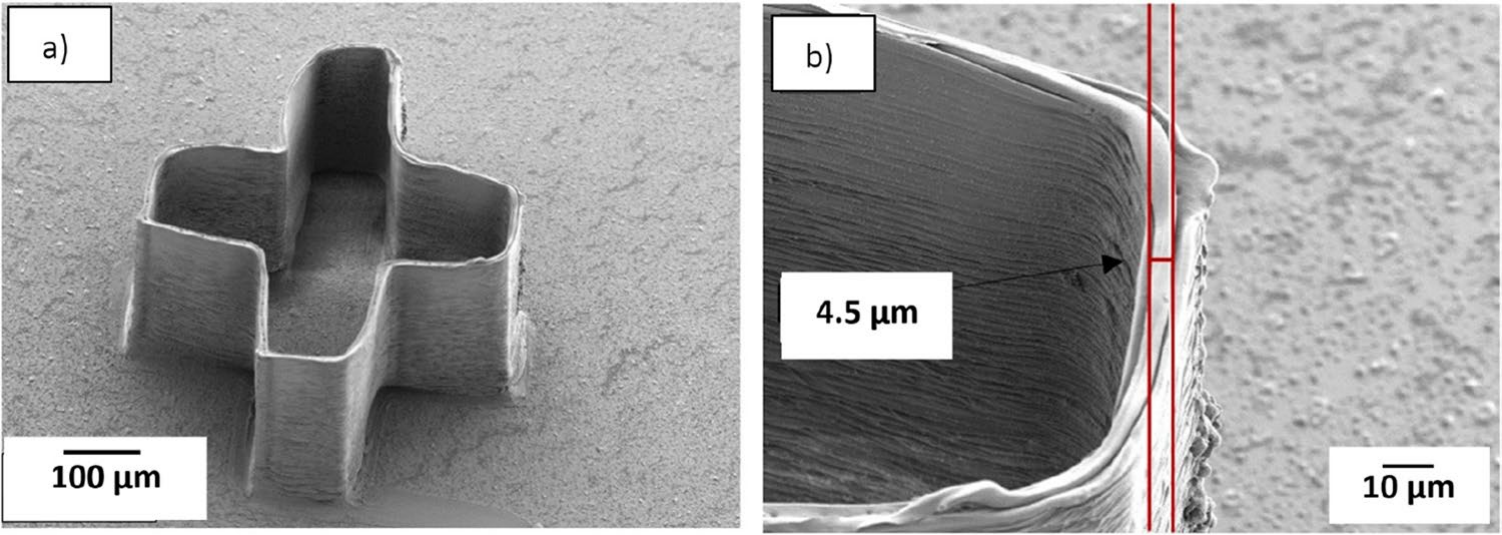
(**a**) The SEM image of 3D printed cross-shaped structure with more than 300 layers. (**b**) The width of the fiber of the top layer is 4.5 µm. The structure printed with long taper 3–9 capillary and ink with 10% (50 kDa).

Test structures according to design 3 (a) were printed to study if it was possible to print several hundred layers and one example is presented in Fig. 10a. The 3–9 capillary that was used for this print was a silane coated capillary, and the ink used for printing these structures was 10% CA (50 kDa). The movement of the glass substrate in the Z direction was 1 µm after printing each layer, and the printing speed (speed of specimen table) in the X and Y direction was 0.5 mm/s. Figure 10a shows a 3D printed cross shaped structure where the printing was stopped after printing 300 layers, which results in the structure of about 300 µm height. The result in Fig. 10b show that the width of the printed strand on the top layers is 4.5 µm, which is close to the internal diameter of the capillary. Based on the results presented earlier in this report, narrower strands would be expected with inks having higher molecular weight and higher concentration of CA.

As seen in Fig. 10, the printed corners were rounded rather than sharp. This stems partially from the printer trying to keep a constant print speed while printing. To achieve sharper corners, the printing speed as well as the extrusion rate should be reduced in the X and Y directions.

**Figure 11:**
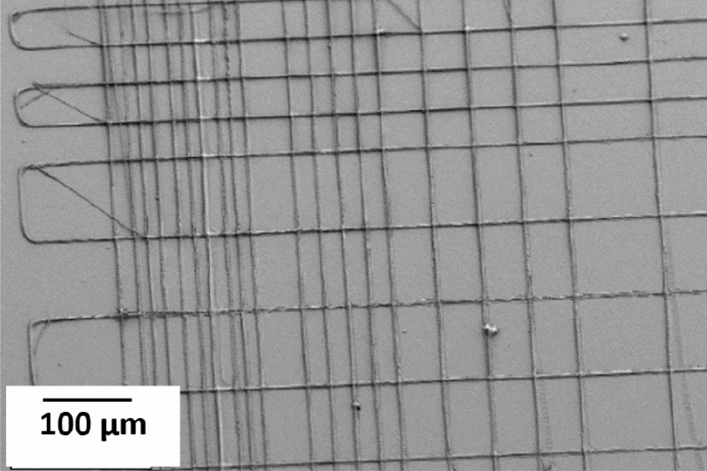
SEM image showing the printing result from the pattern in Fig. 3b, which is printed with a 3–9 capillary and ink with 10% CA (50 kDa).

**Figure 12:**
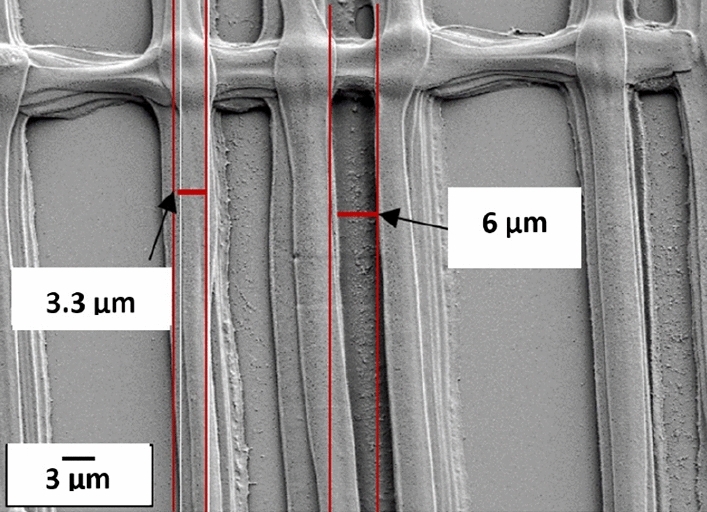
SEM image of the second pattern with measures showing the size of the printed fiber of the top layer, and the smallest printed distance between two adjacent fibers. The 3–9 capillary and ink with 10% CA (50 kDa) were used to print the structure.

The second pattern was designed to see how close it was possible to print two adjacent strands. As explained in Fig. 3b, the pitch between each strand in this design starts from 100 µm and decreases to 10 µm. This design results in a rectangular pattern with aligned strands in parallel, Fig. 11. The 3–9 capillary was used for this structure, and the printing speed in X and Y directions was 0.5 mm/s. For this experiment the ink with 10% CA (50 kDa) was used. Figure 12 shows that the width of the strand of the top layer was about 3.3 µm, which agrees with the internal diameter of the capillary, and indicates that the smallest inter-strand distance obtained was about 6 µm (pitch 10 µm). 5 µm pitch was also evaluated, in addition to the design in Fig. 3b. At this fine pitch the 3 µm strands generally attached to each other, which infers that lateral deflection of the strands has occurred. This is believed to be due to a combined effect from the forces caused by strand wetting and the flexibility of the long-tapered capillaries.

**Figure 13:**
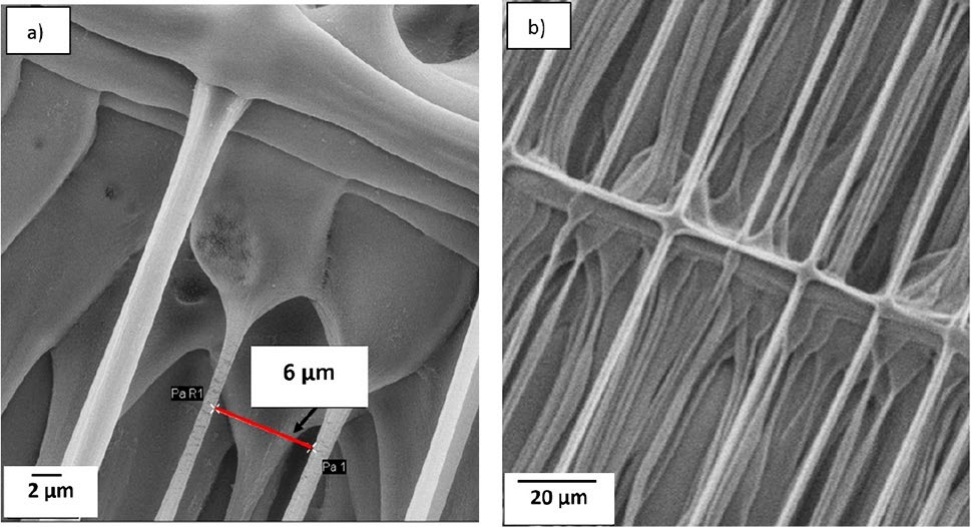
20° tilted SEM results of the third pattern. (**a**) The inter-fiber distance of 6 µm could be found for 10 µm pitches. The 3–9 capillary and ink with 12.5% CA (50 kDa) was used. There is no obvious sagging in the printed strands.

In the second pattern (Fig. 3b), layers were printed on top of each other, creating a wall. The third pattern (Fig. 3c) was used to evaluate printing effects when the printing strands bridge between orthogonal strands. The aim was to print separate strands, and thereby increasing the surface area of the final structure. Figure 13a from the third pattern shows that the smallest printed inter-strand distance observed was 6 µm with 10 µm pitch and the width of the printed stands are 1–3 µm. In this experiment, a 3–9 capillary and ink with 12.5% CA and MW of 50 kDa was used. Figure 13b is showing the same structure at less magnification. The SEM image in Fig. 13 is tilted 20°, and the image indicate that there is normally limited sagging of the printed strands, even though it can be found in some places. As the flexible long tip capillary was used to print these structure we expect that there will be some deflection of the tip due to e.g. wetting forces which would result in laterally curved and displaced strands, as observed in Fig. 13. A lagging of the tip could be observed during the printing, possibly due to viscous drag, and the associated force could then assist in straightening the strands.

## Conclusion

This work shows that it is possible to build cellulose acetate (CA) structures up to 300 layers, corresponding to about 300 µm by the presented DIW technique. Further, the possibility of printing CA strands down to 1 µm in width and lateral inter-strand distances down to 6 µm is also demonstrated. The high detail resolution structures were obtained by controlling the nozzle dimensions and printing parameters as well as the molecular weight and concentration of the CA in the ink. The wetting properties of the ink on both substrate and nozzle in combination with the ink composition have a strong influence on the strand width. When printing membrane-type structures the free-hanging strands will typically have widths similar to, or smaller than, the inner diameter of the nozzle. With the typical wetting of the evaluated inks on bare glass substrates the strand width in the first layer is rather controlled by the outer diameter of the nozzle. The finest inner and outer dimensions of the nozzles produced and used herein, 3 and 9 µm respectively, were two orders of magnitude smaller than those used in earlier studies with cellulose-based inks, facilitating high detail resolution printing. Even though it is possible to reduce the diameter of the printed fiber by increasing the amount and molecular weight of CA in the ink, it should be considered that such changes increase the risk of clogging of the nozzle. In this study, to achieve desired resolution, the printing speed in X and Y directions was 0.5 mm/s, which resulted in extended printing times for larger structures. Looking towards future work, we propose to explore the design of multi-nozzles in analogy with ink-jet printing to enhance the printing speed.

## Data Availability

The datasets used and/or analysed during the current study available from the corresponding author on reasonable request.
